# Stable and tunable MeV *γ*-ray generation via dual-laser inverse Thomson scattering from a laser-plasma accelerator

**DOI:** 10.1038/s41598-026-56639-7

**Published:** 2026-06-16

**Authors:** Hai-En Tsai, Tobias M. Ostermayr, Robert E. Jacob, Qiang Chen, Benjamin J. Greenwood, Robert Ettelbrick, Anthony J. Gonsalves, Kei Nakamura, Liona Fan-Chiang, Ocean Zhou, Sam K. Barber, Fumika Isono, Scott J. Thompson, James T. Johnson, Jay D. Hix, Edward Seabury, David L. Chichester, Carl B. Schroeder, Eric Esarey, Jeroen van Tilborg, Cameron G. R. Geddes

**Affiliations:** 1 Lawrence Berkeley National Laboratory, BELLA Center, Berkeley, 94720 CA USA; 2 University of California, Berkeley, 94720 CA USA; 3 Idaho National Laboratory, Idaho Falls, 83415 ID USA

**Keywords:** Optics and photonics, Physics

## Abstract

Inverse Thomson scattering from laser-plasma accelerators offers a pathway to compact, tunable MeV *γ*-ray sources for reduced-dose radiography and enhanced performance in nuclear resonance fluorescence (NRF)-based isotope identification. However, photon yield and spectral quality are often limited by constraints on interaction geometry and scatter-laser tunability. Here we demonstrate a MeV *γ*-ray source based on a dual-laser inverse Thomson scattering configuration driven by a 100-TW laser-plasma accelerator. Electron beams tunable from 122 to 204 MeV with *<5* mrad divergence and *<1* mrad pointing stability generate *γ* rays with peak energies from 276 keV to 1.2 MeV and yields up to $2\times 10^{7}$ photons per shot. By independently controlling the interaction position and the scatter-pulse duration, we experimentally match the scatter pulse to the walk-off-limited interaction length. Extending the scatter pulse to 200 fs increases photon production by approximately *15%* while maintaining operation in the linear Thomson regime, thereby preserving narrow spectral bandwidth and controlled radiation divergence. Radiographic characterization demonstrates MeV-level penetration and *≈ 0.1* mm spatial resolution, while stable operation is sustained over multi-hour timescales across multiple days. These results show that interaction-length optimization provides a scalable strategy for improving photon yield, spectral control, and operational stability in compact laser-plasma-accelerator-driven *γ*-ray sources.

## Introduction

Inverse Thomson scattering, in which relativistic electrons collide with counter-propagating laser photons, generates tunable MeV *γ*-rays through a double Doppler up-shift process. Compared with bremsstrahlung-based sources^1^, Thomson scattering offers superior spectral tunability, reduced out-of-band dose, and controlled radiation divergence. These characteristics are particularly advantageous for isotope-selective techniques such as nuclear resonance fluorescence (NRF)^2,3^ and for dose-efficient radiographic inspection^4^, where spectral specificity and controlled penetration are essential. Conventional accelerator-based Thomson sources require large-scale facilities and substantial beam-disposal shielding^5^, limiting their deployment in industrial and nonproliferation settings. This motivates the development of compact, application-ready alternatives.

To understand the key parameters governing source performance, the scattered photon energy, bandwidth, and radiation divergence are governed by the electron energy spread $\sigma _e$, divergence $\sigma _{\theta }$, and the scatter-laser strength $a_0$^6^. The photon energy scales as

1 $$\begin{aligned} \hbar \omega _{\gamma } = \frac{2\gamma _e^2(1-\cos \phi )\hbar \omega _L}{1+\gamma _e^2\theta ^2+a_0^2/2}, \end{aligned}$$

where $\gamma _e$ is the electron Lorentz factor, $\omega _L$ the scatter-laser frequency, *ϕ* the collision angle, and *θ* the observation angle relative to the electron beam axis. The normalized vector potential of the scattering laser is $a_0 \simeq 0.86\,\lambda _L[\mu \textrm{m}] \sqrt{I_0[10^{18}\,\mathrm {W/cm^2}]}$. The photon bandwidth can be expressed as

2 $$\begin{aligned} \Delta \omega _{\gamma } = \sqrt{\frac{\gamma _e^4 \sigma _{\theta }^4}{16} +\frac{4\sigma _e^2}{\gamma _e^2} +\frac{a_0^4}{4}}, \end{aligned}$$

and the radiation divergence approximately scales as

3 $$\begin{aligned} \theta _\gamma \sim {\gamma _e}^{-1}{\sqrt{1 + a_0^2/2}}. \end{aligned}$$

**Fig. 1:**
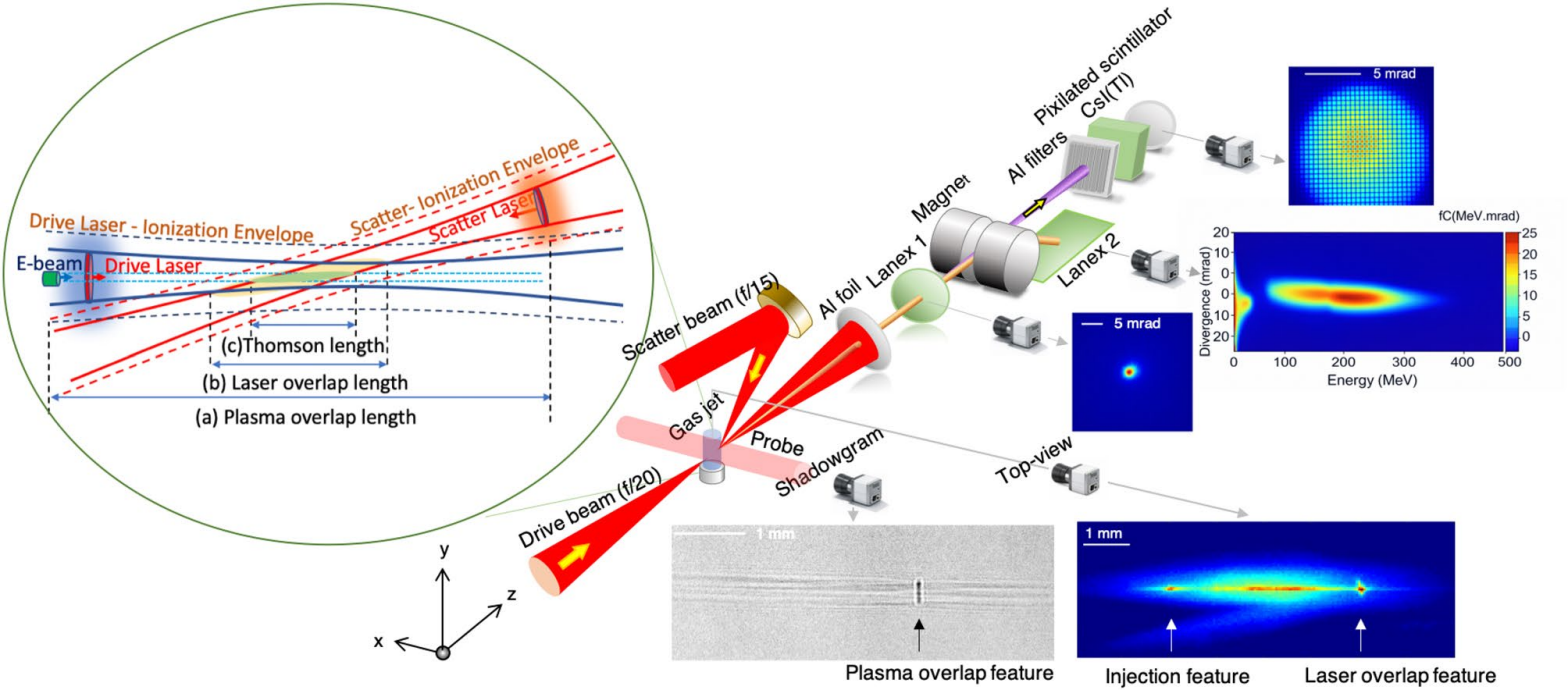
(Color) Layout of dual-laser Thomson scattering experimental setup. The geometry of the nearly-counter-propagating geometry is shown in the inset on the left. Diagnostics for the multi-beam interactions and gamma ray production are implemented to control and optimize the light source.

For fixed electron divergence and energy spread, $a_0$ determines the scattering regime. When $a_0 \gtrsim 1$, relativistic motion of the electron in the field of the scattering laser leads to the nonlinear Thomson scattering regime, resulting in harmonic generation, redshift of the fundamental photon energy (Eq. 1), spectral broadening (Eq. 2), and increased radiation divergence (Eq. 3). In contrast, for $a_0 \ll 1$, the interaction remains in the linear Thomson regime, preserving narrow bandwidth and low divergence.

In addition to the spectral properties, the total photon yield depends on the spatiotemporal overlap of the electron bunch and the scattering laser. The total scattered photon number is given by the space-time integral of the local Thomson scattering rate^7^,

4 $$\begin{aligned} N_{\gamma } = \sigma _T \int v_{\textrm{rel}}\, n_e(t,\textbf{r})\, n_L(t,\textbf{r})\, d^3\textbf{r}\,dt, \end{aligned}$$

where $\sigma _T$ is the Thomson cross section, and $n_e$ and $n_L$ are the electron and photon densities.

In a finite-angle collision geometry, the effective Thomson interaction length (denoted here as the “Thomson length,” $L_T$, green area in Fig. 1 inset) is determined by the spatial overlap of the electron bunch and the focused scattering pulse. It can be estimated as

5 $$\begin{aligned} L_T \sim \min \!\left( Z_R,\; \frac{w_L}{\sin \phi } +\frac{w_e}{\tan \phi }\right) , \end{aligned}$$

where $Z_R$ is the Rayleigh length, $w_L$ and $w_e$ the scattering-laser and electron beam spot diameters, and *ϕ* the collision angle. For Gaussian transverse electron and laser profiles with rms sizes $\sigma _e$ and $\sigma _L$, Eq. (4) can be approximated as

6 $$\begin{aligned} N_\gamma \simeq \frac{\sigma _T N_e N_L}{2\pi (\sigma _e^2+\sigma _L^2)}\,\eta , \end{aligned}$$

where $N_e$ is the total number of electrons, $N_L$ is the total number of photons in the scattering pulse, and *η* is a longitudinal overlap efficiency factor. For a Gaussian laser profile with duration $\tau _L$, the overlap efficiency can be written as

7 $$\begin{aligned} \eta = \operatorname {erf}\!\left[ \sqrt{\ln 2}\, \frac{L_T}{c\tau _L}\right] , \end{aligned}$$

which represents the fraction of the laser pulse that overlaps the interaction region. In the short-pulse limit ($c\tau _L \ll L_T$), $\eta \rightarrow 1$. For longer pulses ($c\tau _L \gtrsim L_T$), the overlap decreases approximately as $L_T/(c\tau _L)$. For a fixed scatter-pulse energy, increasing the pulse duration reduces the peak photon density ($n_L \propto 1/\tau _L$), while the total photon number $N_L$ remains approximately constant. As a result, the photon yield is primarily governed by the overlap efficiency *η*. In the present regime, both the characteristic electron radiation (depletion) length and the laser depletion length are much longer than $L_T$, indicating that the interaction is limited by the Thomson length instead of energy depletion. Rather than increasing the peak intensity—which would drive the interaction toward the nonlinear regime—an alternative strategy is to extend the scatter-pulse duration to match the Thomson length. This approach maximizes the overlap efficiency while maintaining $a_0 \ll 1$, thereby preserving narrow spectral bandwidth and controlled radiation divergence^8^.

Laser-plasma accelerators (LPAs)^7,9,10^ provide relativistic electron beams over centimeter-scale distances using ultra-intense femtosecond lasers. Over the past two decades, LPAs have reached energies from hundreds of MeV^11–18^ to multi-GeV regimes^19–25^, enabling compact all-optical Thomson sources^26,27^. LPA-driven Thomson sources have been demonstrated in plasma-mirror and dual-laser configurations. Plasma-mirror schemes provide intrinsic overlap^28–32^ but limited parameter flexibility. Dual-laser geometries enable independent optimization of drive and scatter pulses^33–36^; however, prior demonstrations primarily increased photon yield by raising laser intensity or electron charge while keeping pulse duration and interaction position fixed, often operating near the nonlinear regime.

In contrast, this work emphasizes systematic interaction control while operating in the linear Thomson regime, thereby maintaining narrow spectral bandwidth and divergence. Using a 100-TW dual-laser system with independent drive and scatter pulses, we implement longitudinal interaction-position optimization and controlled extension of the scatter-pulse duration to match the Thomson length. The scatter pulse is optimized to 200 fs (FWHM), enhancing photon production by 15 *%* without compromising spectral quality or increasing radiation divergence. We further demonstrate sustained, facility-style operation over multi-hour timescales, comparable to recent 100-TW LPA-based free-electron-laser performance^16^. Radiographic characterization confirms MeV-level penetration and 0.1 mm spatial resolution, consistent with prior simulations^37^. These results establish a scalable pathway toward enhanced radiographic performance and improved photonuclear diagnostics, including photofission and NRF.

In the following Results section, we present stable electron-beam generation from 122 to 204 MeV, plasma-based overlap diagnostics, *γ*-ray characterization, and spatial cross-correlation measurements validating interaction optimization. Radiographic characterization confirms MeV-level penetration, narrow divergence, and long-term stability. Technical details are provided in the Methods section.

## Results

**Fig. 2:**
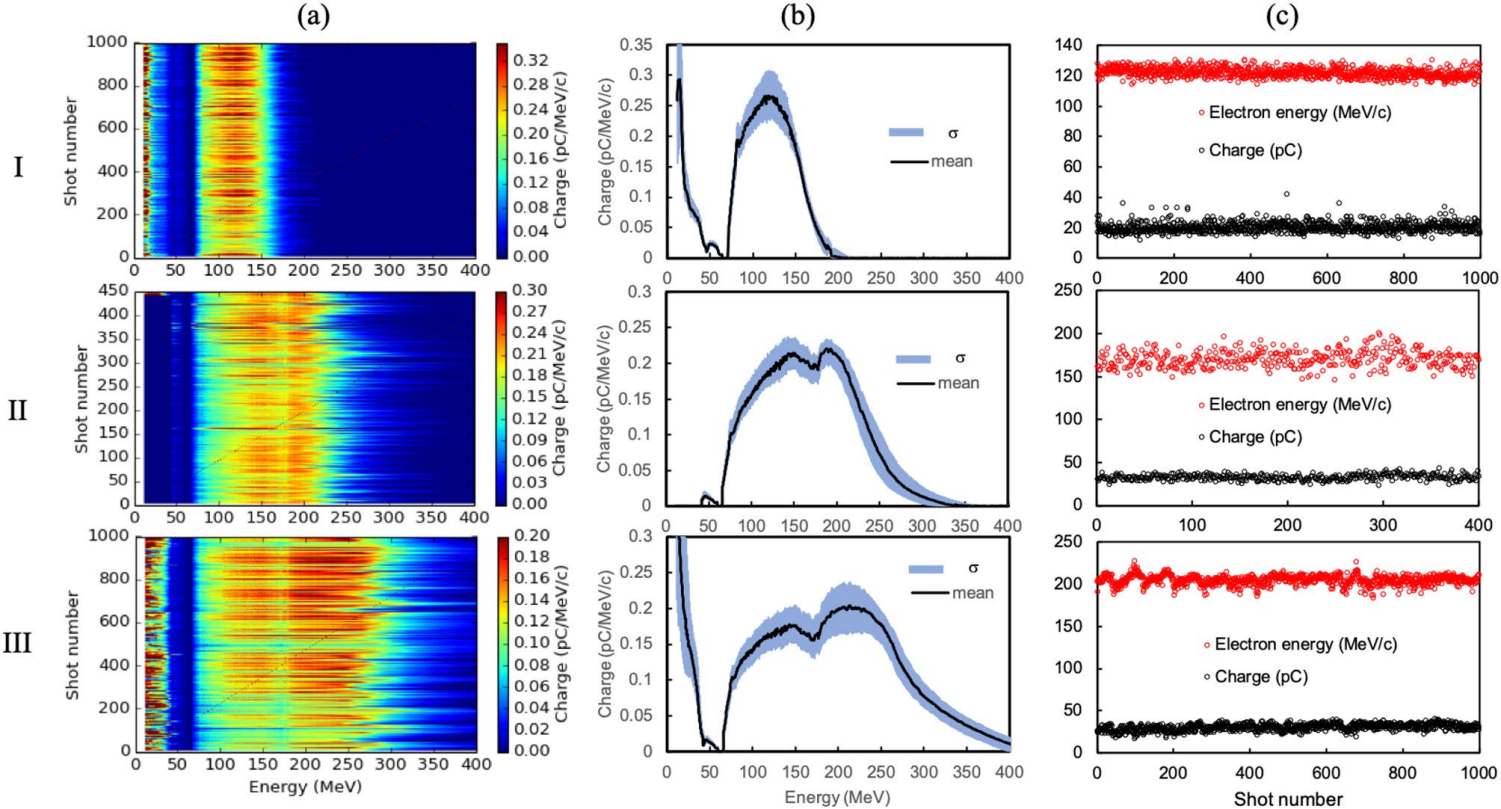
The waterfall plots (**a**) and averaged electron spectra (**b**) of hundreds or 1000 consecutive shots with statistic plots (**c**) showing charge and energy stability. These e-beams were generated via ionization injection in helium gas doped with *0.5 %* nitrogen in three conditions: row I, II, and III producing stable energy, charge, divergence, and pointing as statiscs shown in Table1.

**Table 1:** Comparison of average energy $E_e$, charge $\rho _e$, FWHM divergence $\theta _e$, and rms pointing for three LPA conditions.

Condition	Energy $E_e$ [MeV]	Charge $\rho _e$ [pC]	FWHM$\theta _e$ [mrad]	rms pointing [mrad]
I	*122± 3*	*20± 3*	*5.5± 1.1*	1.2
II	*170± 10*	*33± 3*	*3.5± 0.2*	0.9
III	*204± 6*	*30± 4*	*3.2± 0.2*	0.9

### Stable electron-beam generation

The LPA was operated using ionization injection in helium gas doped with 0.5% nitrogen under multiple stable operating conditions. By adjusting the drive-laser focus position, plasma density, jet height, laser pulse duration, and laser energy, three reproducible electron-beam modes were established. Figure 2 presents representative waterfall spectra and statistical distributions obtained from 450 to 1000 consecutive shots.

The electron-beam parameters for the three operating conditions are summarized in Table 1. The spectra exhibit a low-energy tail (*<50* MeV) and a dominant peak with mean energies ranging from 122 to 204 MeV. The corresponding beam charges are 20–33 pC with FWHM divergences of approximately 3–6 mrad. All operating modes demonstrate stable performance with rms pointing below 1.2 mrad.

Operating below the dephasing limit, increasing the effective plasma length resulted in higher mean electron energies. The plasma interaction lengths were approximately 4.5 mm, 5.6 mm, and 6.5 mm, determined by the gas-jet heights for conditions I, II, and III, respectively. Under these conditions, the LPA delivered thousands of consecutive shots with divergence *łe 5* mrad and pointing jitter below 1.2 mrad. This controlled beam transport prevented electron interception at the 40-mm-diameter beam pipe located 2 m downstream, thereby minimizing bremsstrahlung background prior to scintillator-based diagnostics.

### Plasma-based spatial and temporal overlap diagnostics

**Fig. 3:**
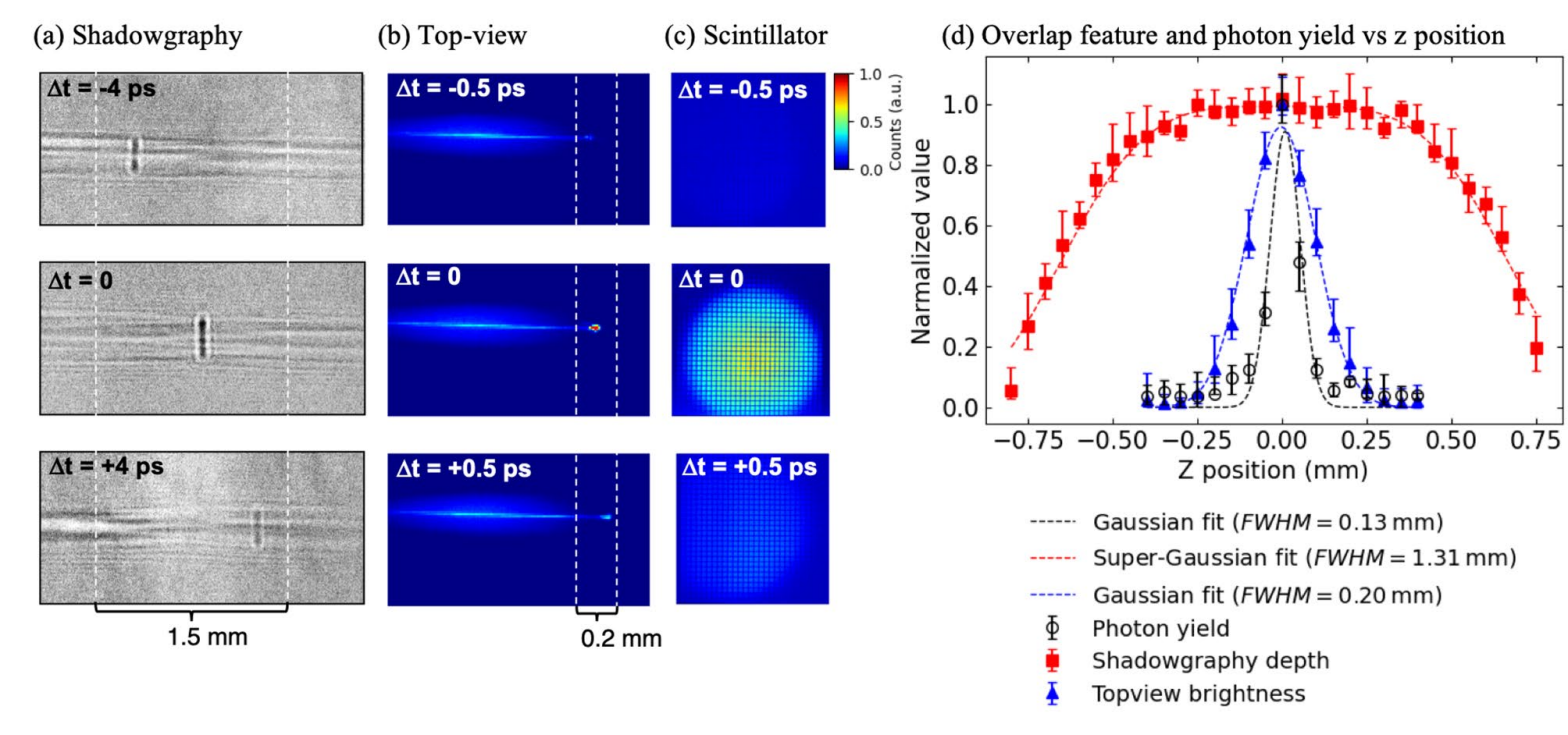
(**a**) Transverse probe shadowgraphy images showing plasma channels driven by the drive (from left) and scatter (from right) pulses. A vertical overlap feature marks the pulse collision as the scatter delay is scanned from *-4* to *+4* ps; its depth is evaluated within the 1.5 mm window indicated by the dashed lines. (**b**) Top-view images at representative scatter delays from *-0.5* to *+0.5* ps, with the overlap brightness evaluated within a *∼*0.2 mm window (dashed lines). (**c**) Scintillator images showing photon yield at scatter delays from *-0.5* to *+0.5* ps. (**d**) Shadowgraphy depth, top-view brightness, and photon yield as functions of the longitudinal position *z*. The position of maximum depth and brightness defines *z=0*, at which the photon yield is also maximized. Gaussian and super-Gaussian fits yield FWHM values of 1.31 mm (shadowgraphy depth), 0.20 mm (top-view brightness), and 0.13 mm (photon yield).

To achieve precise spatial and temporal alignment between the drive pulse, scatter pulse, and electron bunch, complementary plasma diagnostics—namely transverse probe shadowgraphy and top-view imaging—were employed, as illustrated in Fig. 1 and its inset, where three characteristic overlap lengths are defined. (**a**) The *plasma overlap length* arises from the transverse extent of the plasma envelopes generated by each laser pulse, which extend several times beyond the focal spot size ($w_{\textrm{FWHM}}=20~\mu$m). Approximating each plasma volume as a cylindrical region with a diameter of $\sim 250~\mu$m, the transverse projection of two such volumes intersecting at $160^\circ$ yields an expected plasma overlap length of *∼ 1.5* mm, comparable to the Rayleigh length of *∼ 1.2* mm. This overlap is diagnosed using transverse probe shadowgraphy. (**b**) The *laser overlap length* is defined by relativistic laser-laser beating within the plasma envelope and is diagnosed via nonlinear plasma emission observed in top-view imaging. A 400-nm bandpass filter is used to isolate the second-harmonic emission arising from relativistic electron oscillation, enabling selective observation of this overlap. The resulting emission is localized to a window of *∼ 0.2* mm. (**c**) The *Thomson length* is determined by the spatial overlap between the accelerated electron bunch (with transverse size $\sim 15~\mu$m at the interaction) and the focused scatter-laser spot ($w_{\textrm{FWHM}}=20~\mu$m), which defines the effective interaction region for photon generation. This overlap corresponds to a longitudinal length of *∼ 0.1* mm.

Figure 3 demonstrates how these overlap conditions are experimentally resolved. In Fig. 3a, transverse shadowgraphy images show a vertical plasma overlap feature that shifts longitudinally as the scatter delay is scanned from *-4* to *+4* ps. The center of the shadowgraphy depth profile is defined as *z=0* and corresponds to zero scatter delay. Figure 3b presents top-view images at representative scatter delays from *-0.5* to *+0.5* ps, where the second-harmonic emission spark peaks at the same position, *z=0*, and at zero scatter delay. Figure 3c shows scintillator images of the corresponding *γ*-ray photon yield recorded at the same scatter delays, with the photon yield also peaking near *z=0*. Together, these measurements demonstrate a robust technique for achieving and diagnosing spatial and temporal overlap, in which the interaction region is progressively confined from the millimeter-scale plasma overlap length to the sub-millimeter laser and Thomson overlap lengths.

Gaussian and super-Gaussian fits to the shadowgraphy depth, top-view brightness, and photon yield distributions yield FWHM values of 1.31, 0.20, and 0.13 mm, respectively. These values are in good agreement with geometric estimates based on the overlap lengths defined in the inset of Fig. 1, confirming the consistency between the diagnostic measurements and the expected interaction geometry.

### MeV *γ*-ray generation and detection

We present beam-profile measurements, spectral characterization, interaction optimization, and stability assessment of the Thomson-scattered *γ*-ray source.

**Fig. 4:**
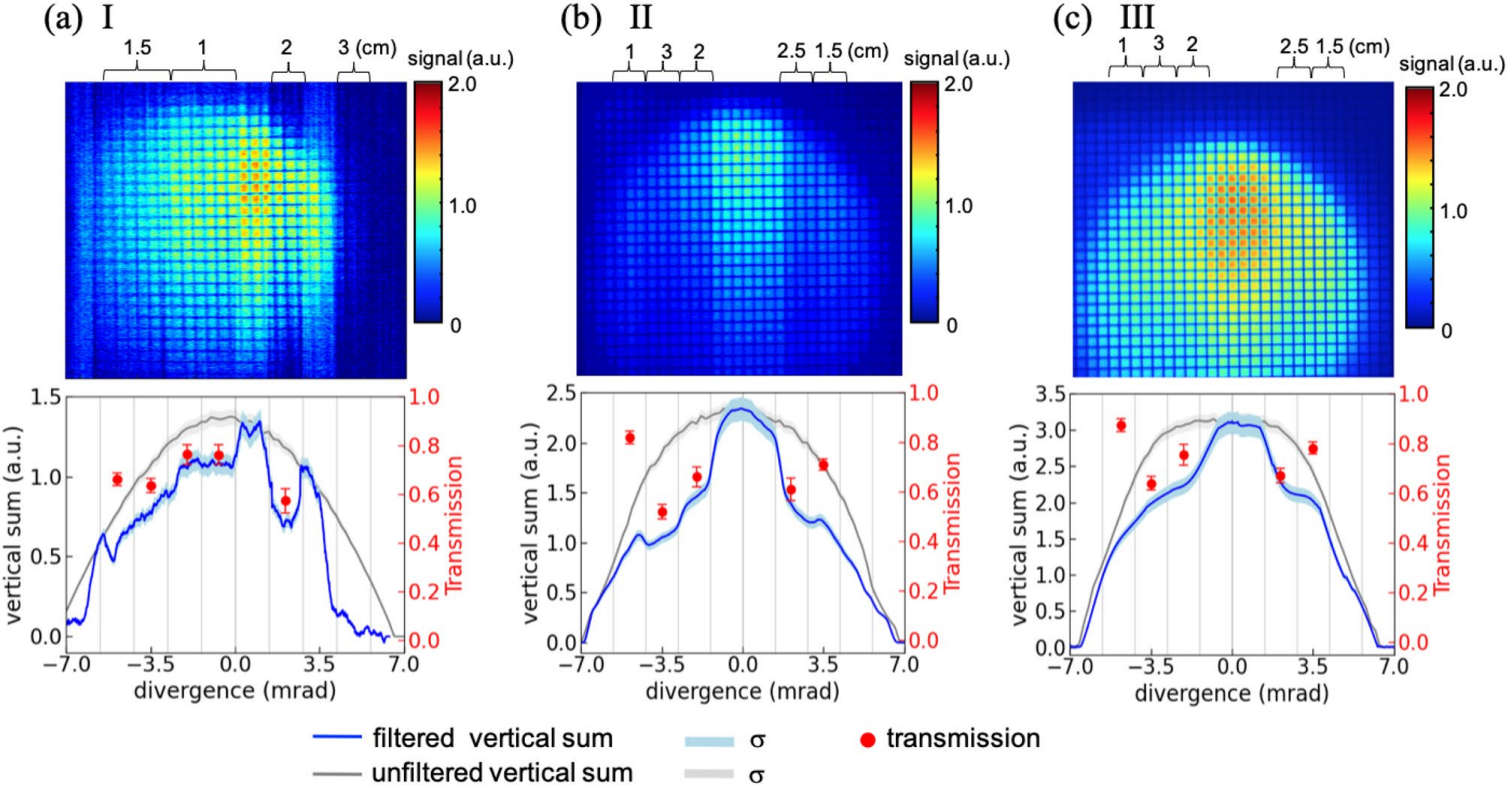
Thomson scattering from electron beams with energies of (**a**) 122 MeV (condition I), (**b**) 170 MeV (condition II), and (**c**) 204 MeV (condition III). *Top:* Twenty-shot-averaged *γ*-ray images recorded on a pixelated CsI(Tl) scintillator after transmission through aluminum filters of varying thicknesses (labeled above each image). *Bottom:* Horizontal lineouts of the vertically integrated *γ*-ray signal (dark blue curves) as a function of divergence angle (*θ*), with extracted transmission values shown as red markers. Shaded light-blue bands indicate the standard deviation over 20 shots. Gray curves represent the corresponding vertically integrated lineouts from unfiltered reference images. Only transmission data within a *± 5* mrad acceptance are used for photon-energy evaluation.

**Fig. 5:**
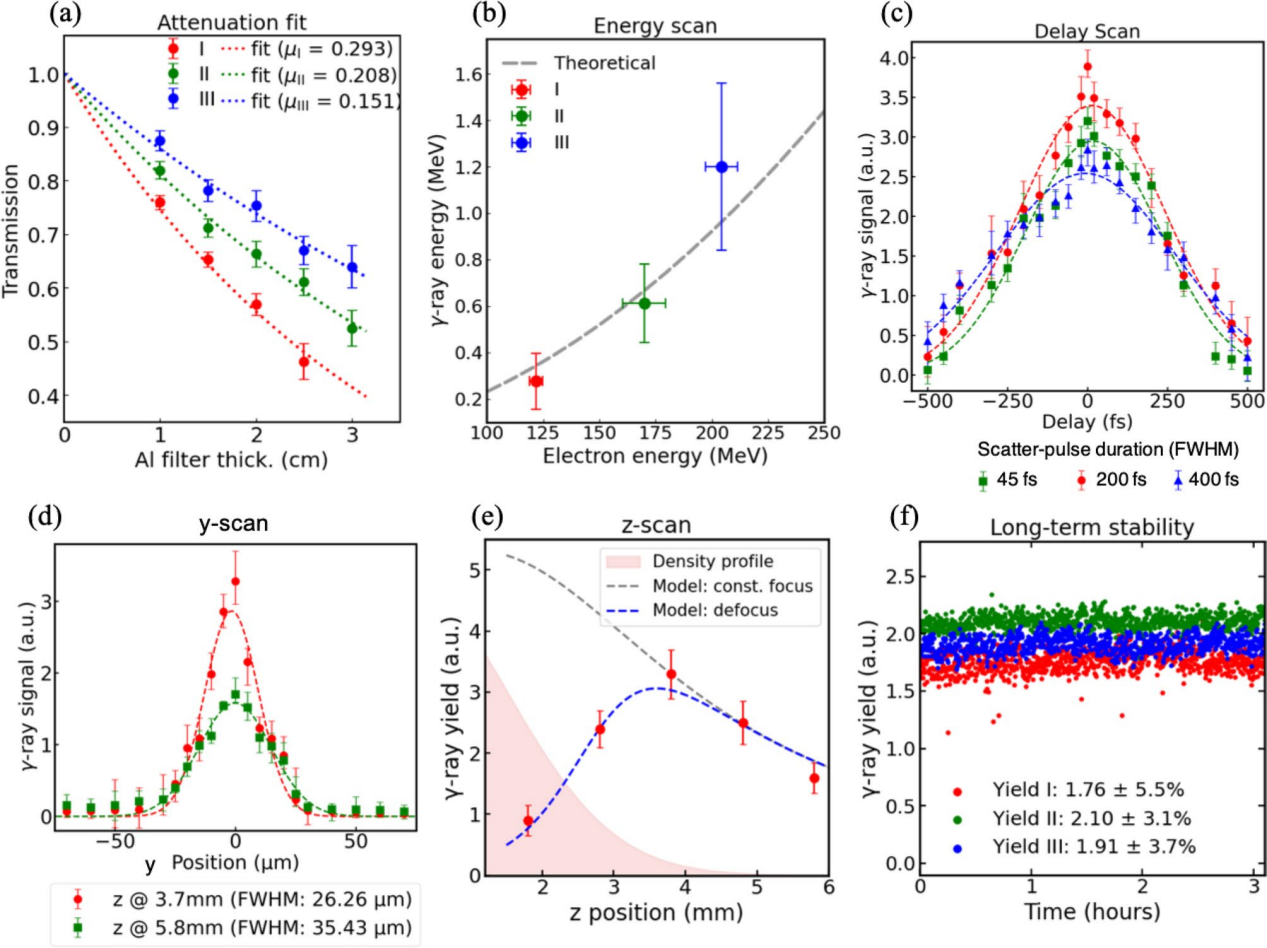
(**a**) Measured *γ*-ray transmission as a function of aluminum filter thickness for experimental conditions I, II, and III (red, green, and blue symbols, respectively). The data are fitted with exponential attenuation models, $T(x)=\exp (-\mu x)$ (dotted lines), yielding attenuation coefficients $\mu _{\textrm{I}}$, $\mu _{\textrm{II}}$, and $\mu _{\textrm{III}}$. The corresponding effective photon energies of 276 keV, 617 keV, and 1.2 MeV are inferred using aluminum mass attenuation coefficients from the NIST database. (**b**) Retrieved *γ*-ray energies for conditions I, II, and III as a function of electron energy. The gray dashed line indicates the theoretical prediction based on the Thomson scattering formula [Eq. (1)]. (**c**) *γ*-ray yield as a function of scatter delay for three scatter-pulse durations: 45 fs (green), 200 fs (red), and 400 fs (blue). Dashed curves represent Gaussian fits with FWHM values of 503 fs, 538 fs, and 656 fs, respectively. (**d**) Electron transverse size measurement inferred from the *γ*-ray yield as a function of the vertical position *y* of the scatter-laser focus. Data are shown for two interaction positions relative to the gas-jet center: 3.7 mm (red) and 5.8 mm (green), with Gaussian-fit FWHM values of 26.3 *μ*m and 35.4 *μ*m, respectively. **(e)** *γ*-ray yield as a function of interaction position along the laser propagation direction (*z*-scan), showing an optimal scattering location. Model curves illustrate the expected yield trend with (blue) and without (gray) laser-plasma refocusing effects at the gas-jet exit**(f)** *γ*-ray yield measured over multiple hours, demonstrating long-term stability with rms fluctuation within $\pm 5\%$rms across three experimental runs.

#### *γ*-ray spatial beam profile

We first characterize the spatial distribution and shot-to-shot stability of the generated *γ*-ray beam. For *γ*-ray detection, a pixellated CsI(Tl) scintillator array was positioned 5 m downstream of the interaction point, and the scintillation emission was recorded by a digital camera. The detector has an active area of $70~\textrm{mm} \times 70~\textrm{mm} \times 40~\textrm{mm}$, consisting of a *30 × 30* pixel matrix with individual pixel dimensions of $2~\textrm{mm} \times 2~\textrm{mm} \times 40~\textrm{mm}$. As shown in Fig. 1 (upper right), this configuration covers forward radiation within a *∼ 14* mrad acceptance angle. An example of an unfiltered 1.2 MeV *γ*-ray beam is presented. To ensure detection of Thomson-scattered photons while minimizing bremsstrahlung background, the electron-beam divergence (*∼ 5* mrad) and pointing variation (*∼ 1* mrad rms) were maintained well below the acceptance of the downstream magnetic spectrometer (*∼ 20* mrad rms), preventing interception of beamline components. Bremsstrahlung radiation from beamline interactions can otherwise exceed the Thomson signal by a factor of 10–20. Blocking either the electron beam or the scattering laser reduced the measured *γ*-ray signal to the background level, confirming Thomson scattering as the origin of the emission.

After background subtraction and spatial smoothing, near-Gaussian or super-Gaussian beam profiles were obtained. The gray curves in Fig. 4a–c, bottom row, show the vertically integrated profiles of three unfiltered *γ*-ray beams generated under electron conditions $\textrm{I}$, $\textrm{II}$, and $\textrm{III}$ using a 45 fs (FWHM) scattering pulse with the interaction point located 2.5 mm downstream of the gas-jet center. The measured beam divergence is approximately *∼ 9* mrad (FWHM) in both the vertical and horizontal directions. The *γ*-ray beam was reproducibly generated in approximately 90% of shots, with $\sim 10\%$ dropouts primarily attributed to pointing misalignment. During later source-characterization campaigns, the reproducibility improved to nearly 98% when a longer (200 fs FWHM) scattering pulse was used. The stretched pulse increased the effective interaction window along the collision geometry, making the Thomson interaction less sensitive to relative pointing jitter between the electron beam and the scattering laser. For each parameter setting in the scan, 20 shots were typically accumulated to improve the signal-to-noise ratio.

#### Spectral characterization and energy tunability

We next quantify the photon energy using transmission measurements and demonstrate tunability from the sub-MeV to MeV range. An aluminum filter array consisting of $6.35~\textrm{mm} \times 50~\textrm{mm}$ vertical stripes with thicknesses ranging from 1 to 3 cm in 0.5 cm increments was employed. Figure 4 (top row) shows representative aluminum-filtered *γ*-ray images obtained under the same experimental conditions as the unfiltered case: a 45 fs (FWHM) scattering pulse with the interaction position 2.5 mm downstream of the gas-jet center under conditions I, II, and III. Each image represents a 20-shot average of the transmitted *γ*-ray signal, with filter thicknesses labeled above the scintillator images. The filter configuration was slightly adjusted between conditions to accommodate different photon energies. The bottom row of Fig. 4a–c presents the vertically integrated *γ*-ray profiles (dark blue curves) as a function of divergence angle. Transmittance values were extracted for each filter stripe (regions between gray vertical lines) by comparing the filtered lineouts to an unfiltered reference (gray curves) obtained from corresponding 20-shot averages. Error bars (light blue and gray shading) represent the standard deviation of shot-to-shot fluctuations.

For transmission-based spectral inference, the analysis is restricted to a *± 5* mrad half-angle acceptance (both horizontal range and vertically integrated signal). This acceptance is comparable to, but slightly larger than, the characteristic forward emission half-angle ($\sim 1/\gamma \approx 2.5$–4.2 mrad for 204–120 MeV electrons). Although the photon energy decreases with angle according to Eq. 1, the angular intensity distribution is strongly forward-peaked, $I(\theta )\propto (1+\gamma ^2\theta ^2)^{-2}$, such that photons emitted at larger angles contribute progressively less to the detected signal. After accounting for the measured electron divergence (3–5 mrad), averaging the angular-dependent photon energy over a *± 5* mrad aperture yields a mean photon energy only *∼ 15*–20% lower than the on-axis value. The inferred spectral dependence varies only weakly for aperture sizes between *± 4* and *± 6* mrad. The *± 5* mrad acceptance therefore provides a practical compromise between maximizing photon statistics and minimizing angular spectral dependence.

Figure 5a shows the measured attenuation as a function of aluminum thickness. Exponential fits yield attenuation coefficients (see Methods) of $\mu _{\textrm{I}} = 0.293 \pm 0.03$, $\mu _{\textrm{II}} = 0.208 \pm 0.03$, and $\mu _{\textrm{III}} = 0.151 \pm 0.03$. Using aluminum mass attenuation coefficients from the NIST database, these correspond to *γ*-ray center energies of *0.28 ± 0.08* MeV, *0.61 ± 0.15* MeV, and *1.2 ± 0.35* MeV, respectively. Although the transmission-based inference yields an uncertainty of $\sim \pm 0.3$ MeV, the central *γ*-ray energy increases systematically from *∼ 0.3* MeV to *∼ 1.2* MeV as the LPA electron energy is tuned from 121 MeV (*γ =236*) to 204 MeV (*γ =400*), as shown in Fig. 5b. The measured *γ*-ray energies are consistent with predictions from linear Thomson scattering [Eq. 1] using the measured electron energies and the $160^\circ$ collision geometry. Horizontal error bars represent the standard deviation of the electron energy over 20 shots, while vertical error bars indicate the uncertainty in the inferred *γ*-ray energy.

#### Transverse overlap optimization and electron size measurement

Precise transverse overlap between the electron beam and the scattering pulse is essential for maximizing photon yield and extracting source parameters. The *γ*-ray yield was optimized by adjusting the spatial and temporal overlap between the electron beam and the scattering pulse. Figure 5d shows the *γ*-ray yield as a function of the vertical position of the scattering-laser focus at two interaction locations, 3.7 mm (red) and 5.8 mm (green) downstream of the gas-jet center. The measurements were performed under condition I using a 45 fs (FWHM) scattering pulse. Each data point represents a 20-shot average. The dashed curves are Gaussian fits with FWHM values of 26.3 *μ*m and 35.4 *μ*m at interaction positions of 3.7 mm and 5.8 mm, respectively.

The transverse electron-beam size at the scattering plane was inferred from spatial cross-correlation analysis^38–40^,

$$\sigma _y^2 = \sigma _e^2 + \sigma _L^2,$$

where $\sigma _e$ and $\sigma _L$ denote the transverse sizes of the electron beam and the scattering-pulse focal spot, respectively. This method allows simultaneous optimization of photon yield and quantitative determination of the electron-beam size at the interaction location. With a measured scattering spot size of $\sigma _L = 21 \pm 1~\mu$m (FWHM), the deconvolved electron-beam sizes were determined to be $\sigma _e = 15.8 \pm 3~\mu$m and $28.5 \pm 3~\mu$m (FWHM) at 3.7 mm and 5.8 mm, respectively, assuming a Gaussian transverse profile.

These values are consistent with the independently measured electron-beam divergence of *∼ 5* mrad, which predicts transverse expansion over several millimeters of free-space propagation. Previous studies have reported sub-micron ($\ll 1~\mu$m) electron source sizes at the exit of the laser-plasma accelerator^41^. In the present experiment, however, the scattering interaction occurs several millimeters downstream of the gas-jet center. The measured transverse dimensions therefore reflect beam expansion after acceleration rather than the intrinsic source size at injection, and are consistent with transverse growth during several millimeters of free-space drift of a few-mrad divergent beam. This validates the inferred interaction spot size and indicates the potential for improved sub-micron resolution in future implementations with the interaction located at the accelerator exit.

#### Longitudinal interaction-position optimization (z-scan)

The interaction position along the propagation direction (*z*) was optimized for scattering efficiency. Several interaction positions downstream of the gas-jet center were investigated, where the gradually diverging electron beam (average divergence *∼ 5* mrad) spatially and temporally overlaps with the scatter-laser focused to a 21 *μ*m (FWHM) spot. This optimization was performed for electron beams operated under three conditions. As a representative example, Fig. 5e shows measurements obtained using electron beams under condition I with a 45 fs (FWHM) scatter pulse, for which the *γ*-ray yield is maximized at an interaction position located 3.7 mm downstream of the nozzle center. To interpret the observed *z*-dependent yield trend, model calculations are overlaid in Fig. 5e, comparing a constant-focus case with a fixed 21 *μ*m laser spot to a model that accounts for laser defocusing induced by the plasma-density downramp near the jet exit. The plasma-induced defocusing is estimated using a simplified thin-lens model based on the integrated plasma density and applied to the Gaussian beam propagation, as discussed in the Methods section below. The calculations capture the maximum scattering efficiency near the optimal interaction position (*z≈ 3.7* mm), highlighting the combined roles of electron-beam divergence and laser-plasma-induced defocusing in determining the interaction geometry. In contrast, the constant-focus model predicts higher scattering efficiency at lower *z*, motivating future investigation of the plasma-profile shaping near the jet exit.

#### Pulse-duration optimization in the linear Thomson regime

The influence of the scatter-pulse duration on the photon yield was investigated through scatter-delay scans performed at the optimal interaction position ($z = 3.7~\textrm{mm}$), using scatter-pulse durations ranging from 45 fs to 400 fs (FWHM). The pulse duration was adjusted by detuning the compressor while compensating with an optical delay, and was independently measured for each grating separation using a frequency-resolved optical gating (FROG) diagnostic. Figure 5c shows the measured *γ*-ray temporal cross-correlation for representative pulse durations of 45 fs (green), 200 fs (red), and 400 fs (blue). Among the tested cases, the maximum *γ*-ray yield was obtained with a 200 fs pulse, producing a signal approximately 15% higher than that obtained with the 45 fs pulse. The measured correlation window extended to *± 500* fs ($\pm 150~\mu$m), consistent with the *Thomson length* expected from the collision geometry. Gaussian fits to the delay scans yield FWHM of 503 fs (45 fs pulse, green), 538 fs (200 fs pulse, red), and 656 fs (400 fs pulse, blue), in good agreement with the calculated values of 490 fs, 526 fs, and 627 fs, respectively (see Methods: Numerical model of *γ*-ray temporal cross-correlation). The 200 fs scatter pulse therefore provides the best match to the *Thomson length*. This duration corresponds to a $1/e^2$ intensity width of 339 fs and maximizes the longitudinal overlap between the electron bunch and the scatter pulse while avoiding peak-intensity-induced spectral broadening. Under this optimized condition, the scatter-laser peak intensity is estimated to be $3.2\times 10^{17}~\mathrm {W/cm^2}$, corresponding to a normalized vector potential of $a_0 = 0.39$ for a pulse energy of 600 mJ. This intensity enables efficient scattering while remaining within the linear Thomson-scattering regime^35,42–44^. By contrast, calculations based on Eq. (3) indicate that for a 45 fs pulse with $a_0 \sim 1$, the fraction of photons falling within the detector acceptance of *± 5* mrad is reduced by approximately 13% due to increased radiation divergence. This effect further supports the observed optimization (see also Methods: Numerical model of *γ*-ray temporal cross-correlation).

The photon number generated under the optimal condition is estimated to be $2\times 10^7$ for a beam with a central photon energy of 1.2 MeV, using the method described in the Methods section and Eq. 9. The peak brightness is estimated from the measured beam divergence, the photon number distribution inferred from the electron spectrum, the measured source size, and by assuming a 30 fs *γ*-ray pulse duration. This yields a peak brightness on the order of $\mathrm {10^{19}\ photons\ s^{-1}\ mm^{-2}\ mrad^{-2}}$ per *0.1%* bandwidth. Photon production was stable to within $\pm 5.5 \%$ of its average value throughout more than three hours of operation as shown in Fig.5f. The data points represent three different experiments, for which nearly one thousand shots were accumulated for statistical purposes. These measurements were performed under identical electron-beam conditions (I), (II), and (III), without further adjustment or realignment during the operation, demonstrating the reliability of the source for sustained operation in application-relevant radiographic and nondestructive evaluation measurements

**Fig. 6:**
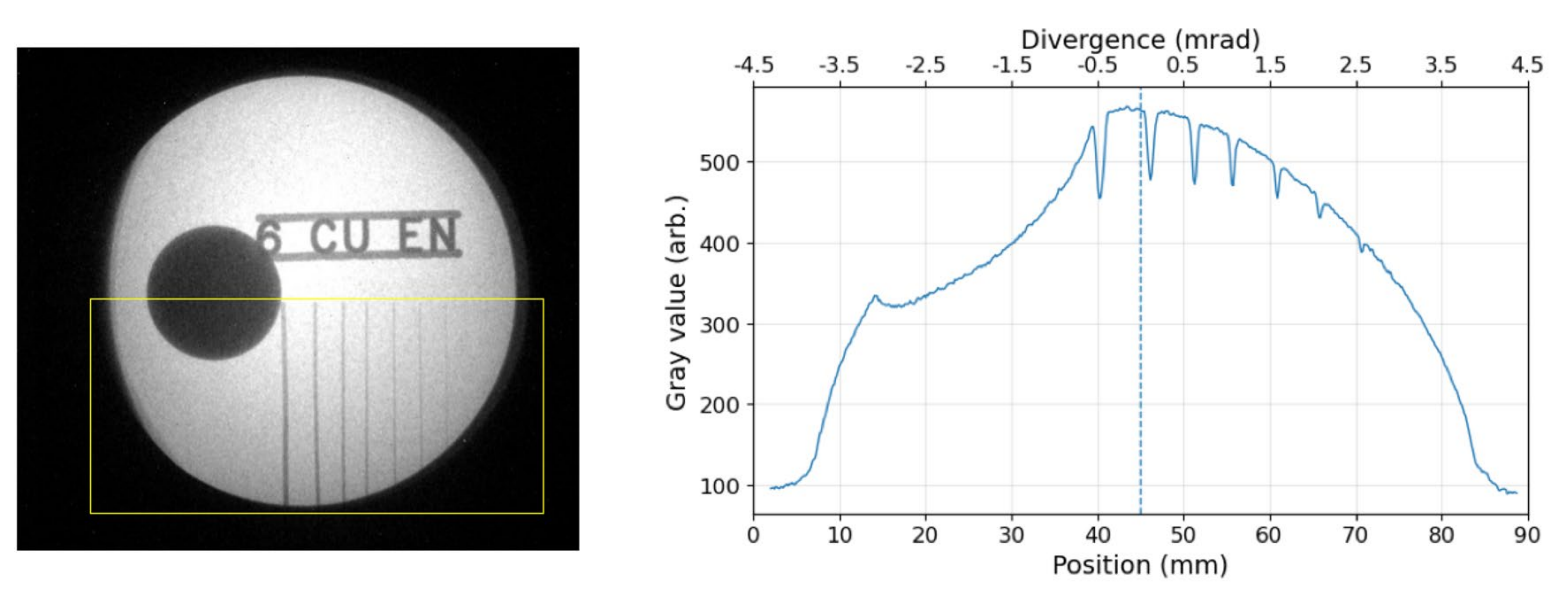
**(Left)** Analysis-level radiographic image of a single-wire copper image-quality indicator and a 1-inch-diameter stainless-steel sphere used as fiducial. **(Right)** Vertical line-sum of the pixel intensities within the region of interest indicated by the yellow box.

#### Radiographic source characterization and application-relevant performance

Radiographic source characterization measurements were performed to evaluate the imaging performance of the source under stable, facility-style operation, with emphasis on parameters relevant to nondestructive evaluation and nonproliferation applications. Absolute dose, beam divergence, and transverse profiles were measured using a compact ionization chamber (RadCal Corp., model 10*×*6–0.6), while spatial imaging and resolution characterization were conducted using a high-dynamic-range digital radiography panel (Novo 22WN, NOVO DR). The detector provides a *42.6 × 35.5* cm active imaging area (2878*×*2350 pixels) with a pixel pitch of 148 *μ*m and a 16-bit dynamic range, positioned 10 m downstream of the source. Using a stretched 200 fs (FWHM) scatter pulse under operating condition III, producing electron energies up to 204 MeV and Thomson *γ*-ray energies of *1.2 ± 0.3* MeV, transverse dose profiling revealed a near-Gaussian beam with a peak dose rate of approximately 32 mR h$^{-1}$ and a divergence of *∼ 7* mrad (FWHM), consistent with independent CsI(Tl) scintillator measurements. The combination of narrow divergence and low dose is a key advantage of Thomson photon sources for high-resolution radiography.

Radiographic imaging was performed using standardized image-quality indicators (IQIs), including single-wire copper targets and a 1-inch-diameter stainless-steel sphere as a fiducial. A total of twelve 175-s exposures were acquired at a 1 Hz repetition rate, and the background-subtracted image is shown in Fig. 6. All seven copper wires are clearly resolved, including the smallest with a diameter of 250 *μ*m, as confirmed by the corresponding vertical lineout. These results demonstrate sub-millimeter spatial resolution at MeV photon energies, a regime that is difficult to access with conventional bremsstrahlung sources at comparable dose levels. Additional measurements using a duplex-wire IQI positioned near the beam port, corresponding to a geometric magnification of approximately 1.7, demonstrated a spatial resolution of 0.13 mm (3.85 line pairs mm$^{-1}$). This resolution is primarily limited by the detector pixel size and exceeds the minimum spatial-resolution requirements specified by ANSI 42.55^45^. Together, these measurements demonstrate that the source characteristics—MeV photon energy, narrow divergence, low dose, and stable multi-hour operation—translate directly into performance metrics relevant for radiographic inspection and nonproliferation applications^46^. These results highlight the potential of LPA-driven Thomson sources for high-contrast imaging of dense objects.

**Fig. 7:**
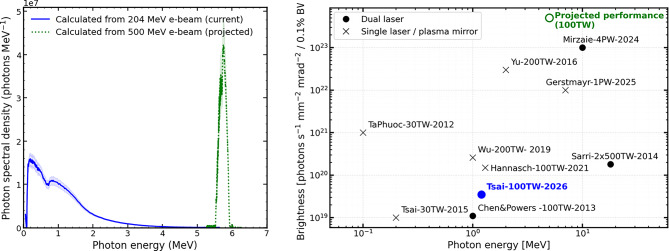
**(Left)** Calculated Thomson spectra for measured condition III using a 204 MeV electron beam (solid blue), compared with a recently demonstrated 500 MeV beam^16^ with $\sim 2\%$ energy spread (dashed green). Spectra are normalized to equal photon yield, illustrating spectral compression and increased peak spectral density. **(Right)** Spectral brightness versus photon energy for representative Thomson/Compton sources. Circles denote dual-laser and crosses denote single-laser/plasma-mirror configurations. The present work (blue) and projected performance based on the 500 MeV beam parameters (green) indicate substantial brightness enhancement at multi-MeV photon energies.

## Discussion

This work demonstrates systematic control of the interaction in a dual-laser inverse Thomson scattering source operating in the linear Thomson regime. A 100-TW dual-laser system is employed to generate stable electron beams with central energies tunable from 122 to 204 MeV. With independent control of the scattering laser, we scanned both the longitudinal interaction position and the scatter-pulse duration. Photon yield was enhanced by 15% by extending the scatter-pulse duration to 200 fs, matching the walk-off-limited Thomson length, rather than increasing peak intensity with the shortest 45 fs pulse and approaching the nonlinear regime. Operating in the linear Thomson regime ($a_0 \ll 1$), the emitted radiation retains the polarization of the incident laser field (here *p*-polarized), while the spectral bandwidth and divergence remain governed by the electron beam and interaction geometry, enabling narrow-bandwidth and low-divergence photon generation. These measurements experimentally validate interaction-length optimization strategies previously proposed in guiding analyses^8^. Alternative interaction geometries, including traveling-wave Thomson scattering^47^, flying-focus schemes^48^, and other structured focusing approaches^49^, have been proposed to extend the effective interaction length.

The longitudinal *z*-scan further reveals the sensitivity of scattering efficiency to collision geometry near the gas-jet exit. Under representative conditions, the photon yield peaks at an interaction position located 3.7 mm downstream of the nozzle center. Model calculations incorporating plasma-induced laser defocusing through a simplified thin-lens description reproduce the observed reduction in yield at lower *z*. The results indicate that the optimal scattering plane results from a balance between tighter electron-beam size at lower *z* and increasing laser focus degradation near the jet exit. These findings emphasize the importance of precise longitudinal interaction control and plasma-profile shaping in compact dual-laser configurations.

The source delivers tunable photons from approximately 0.3 to 1.2 MeV with yields up to $2 \times 10^7$ photons per pulse, together with a significant advance in operational stability. Photon-yield fluctuations remained within $\pm 5.5\%$ (rms) over more than three hours of continuous operation without realignment, with nearly one thousand shots accumulated under identical conditions. Comparable stability was observed across three *γ*-ray operating conditions, demonstrating sustainable 100-TW-class, facility-style LPA photon-source.

Radiographic characterization confirms the penetrative and resolving capability of the MeV-class source. The beam exhibited *∼ 7* mrad (FWHM) divergence and supported spatial resolution at the 0.1 mm level, limited primarily by detector pixel size. Standard image-quality indicators, including 250 *μ*m features, were clearly resolved. These results demonstrate sufficient flux, collimation, and spectral control for nondestructive evaluation and dose-efficient radiography.

Continued improvements in LPA performance further support the development of narrow-band Thomson sources. High-statistics LPA operation at 350–450 MeV with energy spreads of *∼ 5*–10% (FWHM) and predictive sub-percent energy stabilization over $10^5$ shots has been demonstrated^18^. More recently, LPA-driven free-electron-laser operation at *∼ 100* MeV beam energy (tunable to 500 MeV) has achieved energy spreads of *∼ 1*–2% (FWHM), with slice energy spread at or below the 0.1–0.2% level^16^.

Building on this progress, next-stage development of the monoenergetic photon source will target stable operation at $\sim 10^8$ photons per shot using electron-beam parameters accessible to advanced LPA systems, including recently achieved *∼ 500* MeV central energy, $\sim 2\%$ energy spread, $\le 3~\mu$m FWHM source size, and *łe 3* mrad divergence. Under these conditions, the Thomson photon spectrum is expected to extend into the multi-MeV range with substantially reduced bandwidth. The corresponding peak spectral brightness is projected to increase from the present $\sim 3\times 10^{19}$ to $\sim 5\times 10^{23}$ photons s$^{-1}$ mm$^{-2}$ mrad$^{-2}$ per 0.1% bandwidth, as summarized in Fig. 7.

Supporting future isotope-selective interrogation applications will require photon energy spreads at the 2% level. In the linear Thomson limit, where $\Delta E_\gamma /E_\gamma \approx 2\,\Delta E_e/E_e$, this corresponds to percent-level control of the electron energy spread, which is becoming achievable with recent advances in LPA beam quality. In particular, photon energies in the 1–2 MeV range correspond to strong low-lying dipole transitions in actinide isotopes such as $^{235}$U and $^{238}$U^50^. Furthermore, implementation of post-scatter electron deceleration techniques to reduce shielding requirements and background dose for field deployment^51^ reinforces the scalability of compact Thomson sources for next-generation nondestructive assay and nuclear security applications^46^.

## Methods

### Laser system and beam delivery

Experiments were performed using the Hundred Terawatt Thomson (HTT) laser system at the Berkeley Lab Laser Accelerator (BELLA) Center. The Ti:sapphire CPA system delivers dual 800-nm pulses of *40± 5* fs duration at up to 5 Hz. A 4-mJ, 35-fs kHz front-end (Coherent Legend) is split after the common stretcher into driver and scatter arms, amplified in four and three multi-pass stages, respectively, and pumped by a 16-J Thales Gaia laser at 5 Hz to produce 4 J (driver) and 1 J (scatter). A 1-mJ probe beam is extracted from the first driver stage. Both beamlines incorporate independent pulse shaping (Dazzler), expansion, compression, and delay control. Amplified beam diameters are 25.0 mm (driver) and 12.5 mm (scatter), transported through independent *3.3×* expanders and compressors with precise collimation. Pulse durations are measured in situ via FROG and single-shot autocorrelators, reaching 38 fs (driver) and 45 fs (scatter). Both the drive and scattering laser pulses are linearly *p*-polarized relative to the interaction plane. The system operates in a temperature-controlled cleanroom with ultra-stable mounts and active pointing stabilization, achieving *<10~μ*rad (rms) pointing jitter, *<2%* (rms) pulse-energy fluctuation, and *<5%* (rms) pulse-duration variation.

### Laser-plasma accelerator and gas-jet target

For LPA operation (Fig. 1), the driver delivers up to 2.5 J, 38 fs p-polarized pulses focused by an *f=1.5* m (f/20) OAP to a 20 *μ*m (FWHM) spot with an 80% Strehl ratio, corresponding to peak intensities up to $9\times 10^{18}~\mathrm {W/cm^2}$ ($a_0\approx 2$) at the gas-jet entrance. Ionization injection^52–55^ is implemented using a 99.5% He / 0.5% N$_2$ gas mixture, which releases inner-shell electrons near the wake peak to provide robust trapping and high bunch charge. While this approach yields stable, high-charge beams, it typically produces broadband spectra with relative energy spreads ranging from $\sim 10\%$ to 100 *%*, together with increased divergence due to continuous injection and intrinsically higher emittance. The plasma target is a supersonic gas jet formed by a converging–diverging conical nozzle (800 *μ*m throat, 3 mm exit; *M=5*) designed to generate a near flat-top density profile based on fluid-dynamics simulations^56^. Aluminum 3D-printed nozzles were validated by neutral-density interferometry. Gas delivery is provided by a Parker fast solenoid valve operated at 1 Hz with a 2 ms pulse width synchronized with the laser arrival.

### Electron-beam diagnostics

For electron-beam diagnostics, a terbibium-activated gadolinium oxysulfide $\mathrm {(Gd_2O_2S:Tb)}$ phosphor screen is inserted 1.5 meters downstream from the gas jet to detect the electron beam’s spatial profiles on each shot, as shown in Fig.1. Electron-induced fluorescence at 545 nm is imaged by a Basler 12-bit camera. An electromagnetic spectrometer, placed 2 meters downstream from the gas jet, analyzes electron energy on each shot. It consists of an adjustable electromagnet that deflects electrons onto a set of phosphor screens, which are imaged onto four cameras.

### Numerical model of *γ*-ray temporal cross-correlation

**Fig. 8:**
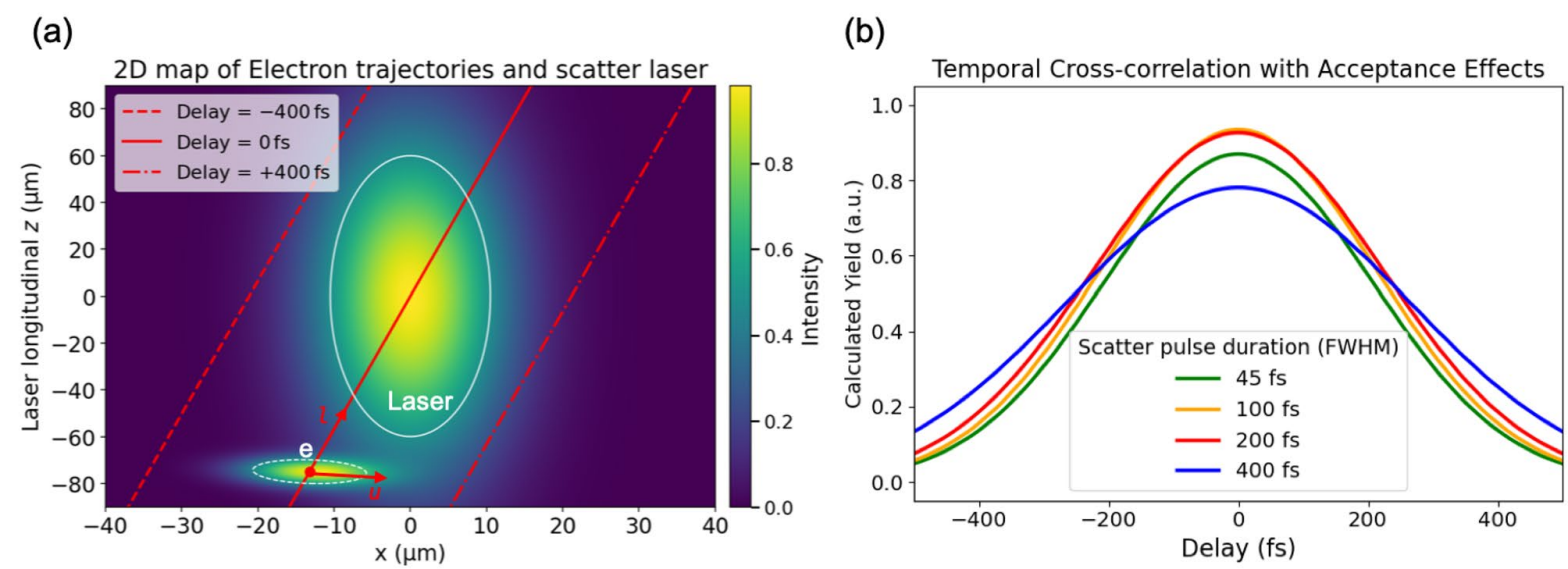
(**a**) 2D Gaussian map in the rest frame of the scattering laser, illustrating an electron bunch traversing the scatter pulse along three relative-delay trajectories (*\ -400* fs, 0 fs, and *\ +400* fs). White contours denote the FWHM of the electron and laser distributions, corresponding to 15 *μ*m and 30 fs for the electron bunch, and 21 *μ*m and 400 fs for the scattering laser, respectively. (**b**) Calculated temporal cross-correlation between the electron and scatter pulses as a function of relative delay, shown for several scatter-laser pulse durations.

To validate the measured temporal cross-correlation of the *γ*-ray signal, we perform a two-dimensional (2D) numerical calculation modeling the spatiotemporal overlap between the electron bunch and the scatter-laser pulse in a $160^\circ$ collision geometry. The calculation is carried out in a reference frame where the *z* axis is aligned with the scatter-laser propagation and the laser pulse envelope is stationary, while *x* denotes the transverse coordinate. In this frame, the electron bunch propagates through the laser at an angle of $10^\circ$ relative to the *z* axis, corresponding to the $160^\circ$ electron–laser collision geometry. We introduce rotated coordinates (*l*, *u*) aligned parallel and perpendicular to the electron trajectory,

$$x = l\sin (10^\circ )-u\cos (10^\circ ), \qquad z = l\cos (10^\circ )+u\sin (10^\circ ).$$

Both the scattering laser and the electron bunch are modeled as 2D Gaussian density distributions,

$$n_L(x,z)=n_{L0}\exp \!\left( -\frac{x^2}{2\sigma _{xL}^2}-\frac{z^2}{2\sigma _{zL}^2}\right) ,\qquad n_e(l,u;t)=n_{e0}\exp \!\left( -\frac{(l-l_c(t))^2}{2\sigma _l^2}-\frac{u^2}{2\sigma _u^2}\right) .$$

As shown in Fig. 8a, the scattering laser has a transverse FWHM of 21 *μ*m and a longitudinal FWHM ranging from 45 fs to 400 fs, corresponding to 13.5–120 *μ*m in the laser reference frame. The electron bunch has transverse and longitudinal FWHM of 15 *μ*m and 30 fs (9 *μ*m), respectively. Here *t* denotes the local interaction time along the electron trajectory (red lines), with $l_c(t)=v\,t$ describing translation along the *l* coordinate. The instantaneous spatiotemporal overlap is quantified by the cross-correlation

$$\begin{aligned} S(t;\tau )=\iint n_L\!\big (x(l,u;\tau ),z(l,u)\big )\,n_e(l,u;t)\,\textrm{d}l\,\textrm{d}u, \end{aligned}$$

where *τ* is the externally applied scatter-laser delay. A finite delay *τ* introduces a transverse trajectory shift

$$\Delta x(\tau )=c\tau \tan (10^\circ ),$$

which selects parallel electron trajectories in the (*x*, *z*) plane, as illustrated by the red lines labeled *± 400* fs and 0 fs in Fig. 8a. The *γ*-ray yield at a given delay is proportional to the time-integrated cross-correlation,

$$\begin{aligned} N_{\gamma }(\tau )\propto \int S(t;\tau )\,\textrm{d}t . \end{aligned}$$

As shown in Fig. 8b, the calculated cross-correlation widths increase from 490 fs to 627 fs as the scatter-pulse duration is extended from 45 fs to 400 fs, in good agreement with experiment. For long scatter pulses (*≳ 300* fs), the peak overlap decreases due to transverse walk-off, which causes the electron bunch to sample regions of reduced spatial overlap.

Finite radiation divergence is incorporated via an acceptance correction based on Eq. (3), assuming an intrinsic electron-beam divergence of 3 mrad and a detector acceptance of *± 5* mrad. The resulting acceptance fractions are 0.87, 0.95, 0.99, and 1.00 for scatter-pulse durations of 45 fs, 100 fs, 200 fs, and 400 fs, respectively, accounting for the higher measured yields at intermediate pulse durations. The 2D approximation is justified because the dominant physics occurs in the transverse–longitudinal plane where angular walk-off is present, while the out-of-plane dimension contributes only a separable Gaussian factor.

### Thin-lens model of plasma-induced scatter-laser defocusing

The scattering laser is modeled as a Gaussian beam with vacuum waist $w_0$ (21 *μ*m FWHM). Plasma-induced defocusing near the jet exit is approximated as an equivalent thin lens with effective focal length $f_{\textrm{eff}}$, determined from the integrated plasma density along the propagation direction,

$$\begin{aligned} \frac{1}{f_{\textrm{eff}}(z)} \propto \int _z^{\infty } \frac{n_e(z')}{n_c\, r_0^2}\, dz', \end{aligned}$$

where $n_e(z)$ is the plasma density profile and z is the longitudinal distance measured downstream from gas-jet center, $n_c$ the critical density, and $r_0$ is a characteristic transverse scale. In this work $r_0 = 30~\upmu$m corresponds to the pre-ionized radius from the axis.

Beam propagation through the plasma lens is evaluated using the complex *q*-parameter formalism. At the lens position the beam parameter transforms as

$$\begin{aligned} q_{\textrm{out}} = \frac{q_{\textrm{in}}}{1 - q_{\textrm{in}}/f_{\textrm{eff}}}, \end{aligned}$$

followed by free-space propagation to the interaction plane. The modified beam radius is obtained from

$$\begin{aligned} w^2(z) = -\frac{\lambda }{\pi \,\textrm{Im}\!\left( 1/q(z)\right) }, \end{aligned}$$

from which the RMS laser size $\sigma _L(z)$ is calculated. The resulting spot size enters the photon-yield scaling,

$$\begin{aligned} N_{\gamma } \propto \frac{1}{\sigma _e^2(z) + \sigma _L^2(z)}, \end{aligned}$$

where $\sigma _e(z)$ is the RMS electron-beam size. This simplified thin-lens model captures the effective focus degradation near the jet exit and reproduces the observed reduction in scattering efficiency at smaller interaction positions.

### *γ*-ray diagnostics

To measure the energy of the generated *γ*-ray photons, a filter-array assembly was placed in front of a pixelated scintillator detector. The filter array consists of nine vertical aluminum strips with thicknesses ranging from 0 to 3 cm. By comparing the *γ*-ray signal recorded behind each aluminum filter with the unfiltered signal on the scintillator images, the transmission for each filter thickness was determined. The measured transmission values were fitted using an exponential attenuation model,

8 $$\begin{aligned} I/I_0 = T(x) = \exp (-\mu x), \end{aligned}$$

where *I* and $I_0$ are the filtered and unfiltered *γ*-ray signal counts, respectively, *x* is the known aluminum thickness, *T*(*x*) is the measured transmission, and *μ* is the effective attenuation coefficient. The photon energy was then inferred by comparing the extracted attenuation coefficients with aluminum mass attenuation coefficients obtained from the NIST database. Figure 5a shows the measured transmission data (symbols) together with the corresponding exponential fits (dashed lines). The total photon number can be estimated using the following equation:

9 $$\begin{aligned} S_{tot} = N_p\cdot \alpha \cdot \epsilon \cdot \theta /4\pi \cdot Q_E, \end{aligned}$$

where $S_{tot}$ is integrated total counts on the CCD which images the scintillator. $N_p$ is the *γ*-ray photon number. *α* is the absorption coefficient of photons by CsI(Tl) scintillator. *ε* is the fluorescence emission from the scintillator. $\theta /4\pi$ is the camera collecting angle. $Q_E$ is the CCD quantum efficiency.

## Data Availability

The datasets generated and analyzed during this study, including *γ*-ray yield measurements, interactionposition scans, and radiographic characterization data, are available from the corresponding author upon reasonable request. The corresponding author is responsible for submitting a competing interests statement on behalf of all authors of the paper. This statement must be included in the submitted article file.
